# Constipation in critically ill patients and its relationship to feeding and weaning from respiratory support

**DOI:** 10.1186/cc12179

**Published:** 2013-03-19

**Authors:** E Spodniewska, A Guha

**Affiliations:** 1 Royal Liverpool University Hospital, Liverpool, UK

## Introduction

Constipation can be defined as a failure of the bowels to open for 3 consecutive days. It is a common problem in intensive care settings but not many studies have so far raised the subject.

## Methods

The audit had the Trust Audit Committee's approval. The existing protocol was used as the benchmark. Patients were studied prospectively to assess compliance with the local bowel protocol, incidence of constipation and relationship to weaning from respiratory support and feeding. All HDU and all mechanically ventilated ICU patients who stayed on the ward for more than 3 days were included, except for patients after bowel surgery and patients with encephalopathy.

## Results

Among the 24 HDU and 21 ICU patients audited in the Royal Liverpool University Hospital, 67% and 57% respectively were constipated. Laxatives were prescribed when patients had not opened their bowels for 3 days in 25% HDU and 75% ICU cases. Taking into consideration that the median age, APACHE II score and length of stay for constipated and nonconstipated patients were similar, the relationship to feeding and respiratory support were assessed. Constipated patients required mechanical ventilation for an average of 6.8 days and nonconstipated for 4.3 days. Failure to feed was observed at least once in 58% constipated and 44% nonconstipated ICU patients and 19% constipated and 12.5% nonconstipated HDU patients.

## Conclusion

There was a high prevalence of constipation among critical care patients with poor adherence to the bowel protocol, which requires improvements in staff awareness and new recommendations (drug chart amendment, and so forth). Duration of mechanical ventilation and failure to feed were greater in constipated than nonconstipated patients. This may indicate a common correlation and further studies are warranted. Due to the possible significant impact of constipation on patients' recovery, each critical care unit should introduce a bowel protocol or ensure compliance with the existing one before the evidence is clearly established.
